# Effects of small-scale temperature shifts on *Aedes* mosquito production and the influence of environmental conditions on quality assessment with the FAO/IAEA flight test device

**DOI:** 10.1051/parasite/2026019

**Published:** 2026-04-10

**Authors:** Wadaka Mamai, Hamidou Maiga, Odet Bueno-Masso, Thomas Wallner, Nanwintoum Sévérin Bimbile Somda, Simran Singh Kotla, Thabo Mashatola, Hanano Yamada, Chantel Janet de Beer, Jérémy Bouyer

**Affiliations:** 1 Insect Pest Control Section, Joint FAO/IAEA Centre of Nuclear Techniques in Food and Agriculture, Department of Nuclear Sciences and Applications, International Atomic Energy Agency P.O. Box 100 1400 Vienna Austria; 2 UMR Mivegec (Maladies Infectieuses et Vecteurs : Écologie, Génétique, Évolution et Contrôle), IRD-CNRS-Univ. Montpellier, Représentation IRD la Réunion – PTU 97495 Sainte Clotilde Cedex La Réunion France; 3 Institut de Recherche Agricole pour le Développement (IRAD) PO Box 2123 Yaoundé Cameroun; 4 Institut de Recherche en Sciences de la Santé/Direction Régionale de l’Ouest (IRSS/DRO), 01 PO. Box 545 Bobo-Dioulasso Burkina Faso; 5 Unité de Formation et de Recherche en Science et Technologie (UFR/ST), Université Norbert ZONGO (UNZ) BP 376 Koudougou Burkina Faso; 6 Centre for Emerging Zoonotic & Parasitic Diseases, National Institute for Communicable Diseases Private Bag X4 2131 Johannesburg South Africa; 7 Wits Research Institute for Malaria, School of Pathology, Faculty of Health Sciences, University of the Witwatersrand Private Bag 3 2050 Johannesburg South Africa; 8 ASTRE, Cirad, INRAE, Univ. Montpellier, Plateforme Technologique CYROI 97495 Sainte‑Clotilde La Réunion France

**Keywords:** sterile insect technique, mass-rearing, climate change, life-history traits, insect flight, *Aedes albopictus*, *Aedes aegypti*

## Abstract

Variations in environmental parameters such as temperature and relative humidity (RH) can significantly affect mass-rearing outcomes and quality assessment. While the impact of broad temperature changes is well documented, the effects of small-scale fluctuations (1–2 °C shifts) on development and quality remain largely unexplored. This study was carried out to investigate the responses of *Aedes aegypti* and *Aedes albopictus* to incremental changes in rearing temperature and to evaluate the influence of temperature and RH on quality assessment with the Food and Agriculture Organization of the United Nations/International Atomic Energy Agency (FAO/IAEA) flight test device (FTD). First-instar larvae were reared under controlled conditions at five constant temperatures (26, 27, 28, 29 and 30 °C) maintaining the larval density and feeding regime as applied in mass-rearing conditions. Key parameters assessed included time to pupation, pupation rate, male escape rate, and body size. Additionally, male *Ae. aegypti* escape rate was evaluated under varying room temperatures and RH levels to assess the consistency and reliability of the FTD. Small-scale temperature fluctuations significantly impacted key life-history traits of *Aedes* mosquitoes, with higher temperatures accelerating time to pupation and enhancing pupation rates in both *Ae. aegypti* and *Ae. albopictus*. Moreover, *Aedes albopictus* consistently exhibited lower pupal yields and male escape rates compared to *Ae. aegypti*. Adult male quality assessed using the FTD was significantly influenced by the environmental temperature, but showed no significant variation in response to changes in RH. These results highlight the importance of precisely controlling environmental conditions during larval mass-rearing and adult quality assessment using the FAO/IAEA FTD to ensure consistent and reliable outcomes.

## Abstract (fr)

Les variations des paramètres environnementaux, tels que la température et l’humidité relative (HR), peuvent influencer de manière significative les résultats d’élevage de masse et l’évaluation de la qualité. Si les effets des variations importantes de température sont bien documentés, l’impact des fluctuations de faible amplitude (1 à 2 °C) sur le développement et la qualité des moustiques reste encore peu exploré. Cette étude a examiné les réponses d’*Aedes aegypti* et d’*Aedes albopictus* à de légères variations de température d’élevage, ainsi que l’influence de la température et de l’HR sur l’évaluation de la qualité à l’aide du dispositif de test de vol développé par la FAO/AIEA. Les larves de premier stade ont été élevées dans des conditions contrôlées à cinq températures constantes (26, 27, 28, 29 et 30 °C), tout en maintenant la densité larvaire et le régime alimentaire appliqués dans les conditions d’élevage de masse. Les principaux paramètres évalués comprenaient le temps de développement larvaire, le taux de pupaison, le taux d’évasion des mâles et la taille corporelle. Par ailleurs, le taux d’évasion des mâles d’*Ae. aegypti* a été mesuré sous différentes conditions de température ambiante et d’HR afin d’évaluer la cohérence et la fiabilité du dispositif de test de vol développé par la FAO/AIEA. Les fluctuations de température, même de faible amplitude, ont significativement affecté les principaux traits biologiques des moustiques *Aedes*. Des températures plus élevées ont accéléré le développement larvaire et augmenté les taux de pupaison chez *Ae. aegypti* comme chez *Ae. albopictus*. En outre, *Ae. albopictus* a présenté de manière constante des taux de pupaison et d’évasion des mâles inférieurs à ceux d’*Ae. aegypti*. La qualité des mâles adultes, évaluée à l’aide du dispositif de test de vol, a été significativement influencée par la température ambiante, mais n’a montré aucune variation notable en fonction de l’humidité relative. Ces résultats soulignent l’importance d’un contrôle rigoureux et précis des conditions environnementales lors de l’élevage larvaire de masse et de l’évaluation de la qualité des adultes à l’aide du dispositif de test de vol FAO/AIEA, afin de garantir des résultats fiables et reproductibles.

## Introduction

*Aedes aegypti* (Linnaeus) and *Aedes albopictus* (Skuse) are two invasive mosquito species of major medical importance, known for transmitting several pathogens responsible for arboviral diseases, including dengue, Zika, chikungunya, and yellow fever. Together, they are responsible for more than 99% of arbovirus transmission to humans [44]. Over the past decade, vector-borne diseases have experienced a significant resurgence in prevalence and severity, with outbreaks of chikungunya and Zika viruses drawing global attention to their increasing public health risk [46]. Despite the existence of some commercially available vaccines for most of these mosquito-borne diseases, their prevention at the global scale continues to depend mainly on controlling mosquito vector populations, interrupting human–vector contact or both.

Abiotic factors such as temperature and relative humidity (RH) are known to play a pivotal role in shaping the biology and ecology of *Aedes* mosquitoes [1, 6, 34, 36, 45]. These variables shape the spatial and seasonal distribution of *Aedes* species and have significant implications for vector management strategies, particularly male-based release methods such as the sterile insect technique (SIT). As poikilothermic and ectothermic organisms, mosquitoes depend on ambient temperature to regulate their body temperature and physiological functions [4, 18], which affect several traits, including development time, synchronization, production, survival, and overall fitness [7]. All these traits are essential for the successful mass-rearing of high-quality mosquitoes in SIT programs. Furthermore, environmental temperature fluctuations can result in asynchronous larval development and unpredictable pupal yield, which in turn affect the accuracy of sex separation [29], ultimately compromising mosquito quality and reducing the overall effectiveness of SIT programs. Extensive research has explored the influence of temperature on mosquito life-history traits, with most studies addressing relatively large temperature shifts, typically 5 °C or more and using larval densities that do not accurately represent those used in mass-rearing settings [8, 10, 20, 38, 40, 43, 48]. Moreover, recent observations from our laboratory, the Insect Pest Control Laboratory (IPCL) and other SIT facilities globally have revealed notable variations in pupal yield and male quality in *Aedes* mosquitoes reared at laboratory temperatures between 26 °C and 30 °C, a range commonly applied or recommended in mass-rearing programs. These observations indicate that even minor deviations in temperature can affect production outcomes, underscoring the need for a more refined understanding of thermal sensitivity in mosquito rearing systems. Importantly, the effects of small-scale temperature fluctuations (e.g., 1–2 °C shifts) on key biological parameters such as larval development, synchronization, pupal yield, and adult fitness remain insufficiently explored. This knowledge gap is particularly critical in the context of mosquito mass-rearing programs that support SIT and other male-based release strategies. Addressing these subtle yet impactful environmental factors is essential for optimizing protocols, improving production efficiency, and ensuring good quality sterile males.

In SIT programs, the biological quality of released sterile males is a key determinant of success [32]. These males must survive, disperse, locate wild females and effectively compete for mating. These performance metrics are traditionally evaluated through longevity studies, mating competitiveness trials in semi-field conditions, and mark-release-recapture (MRR) experiments to assess dispersal [31]. However, these approaches, although important, are often resource-intensive, time-consuming and not suitable for routine quality control. To address these limitations as a routine quality control method, the Food and Agriculture Organization of the United Nations (FAO) and the International Atomic Energy Agency (IAEA) developed a flight test device (FTD) [9] as a rapid proxy to assess male flight ability, a key indicator of male vigor and overall male quality. The FTD is increasingly being implemented in several FAO/IAEA member states to streamline routine quality assessments [14, 22, 23, 37]. However, its efficiency can be influenced by operational and biological factors, highlighting the need for standardization to ensure consistency and reproducibility among experiments and laboratories. Several factors such as male density, age, fan speed, test duration, internal tube color, strain origin, and the addition of olfactory lure have already been investigated for their effect on escape rates [24]. Despite these efforts, the influence of environmental conditions, particularly temperature and relative humidity, on male escape rates in the FTD remains unexplored. These parameters are well-known to influence insect physiology and behavior [6, 16, 33, 42], and could affect mosquito flight activity during FTD testing. It is therefore important to standardize the use of the FTD for routine quality control assessments by selecting optimal temperature and RH ranges, with minimal variation to ensure reproducible and comparable results.

This study was therefore designed with three key objectives: i) to determine whether slight temperature variations, specifically single-step shifts, may affect mosquito larval development, pupal yield and adult quality; ii) to compare *Ae. albopictus and Ae. aegypti* reared under identical rearing conditions, and iii) to evaluate the influence of temperature and RH on male escape rate assessment as measured by the FTD. Enhancing our understanding of how environmental conditions affect both rearing outcomes and quality assessments is essential for refining and harmonizing standard operating procedures (SOPs), thereby ensuring consistent assessment of sterile male quality.

## Methods

### Source of mosquito colonies and maintenance

Experiments were conducted using two well-established *Aedes* mosquito colonies maintained at the Insect Pest Control Laboratory (IPCL), Seibersdorf, Austria. These were: i) the *Ae. aegypti* Brazil strain*,* originally collected from Juazeiro, Brazil, and supplied by Biofabrica Moscamed, an IAEA Collaborative Center, since 2012; and ii) the *Ae. albopictus* Rimini strain, sourced from Italy, provided by the Centro Agricoltura Ambiente, also an IAEA Collaborative Center, since 2018. Both colonies are routinely maintained under mass-rearing conditions at the IPCL using the FAO/IAEA standardized rearing rack unit and mass-rearing cage systems. Larvae were reared in a climate-controlled room at 28 ± 2 °C with 80 ± 10% RH, following developed mass-rearing guidelines [11] with modifications to diet ingredients and feeding regimes, as described in Mamai *et al.* [26]. The adults were maintained at 26 ± 2 °C with 60 ± 10% RH in mass-rearing cages described by Maiga *et al.* [25]. Both rooms operated under a 14:10 h light–dark (L:D) cycle, with 1-hour simulated dusk and dawn periods.

### Effect of temperature on *Ae. aegypti* and *Ae. albopictus* development and quality

**Figure S1.**
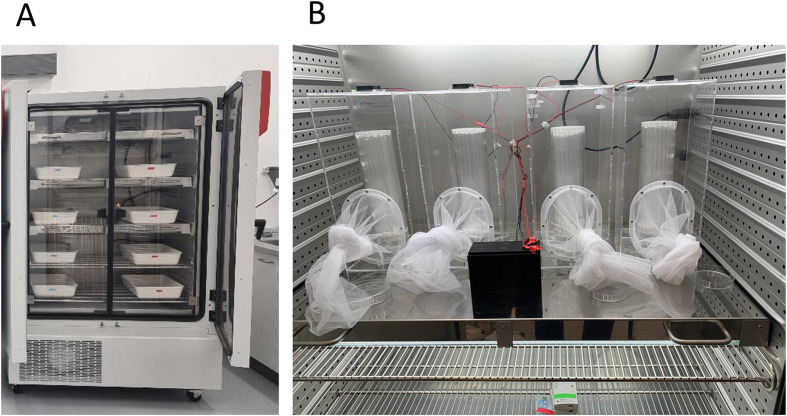
Setting up the climate-controlled chamber (KBWF (E6), model KBWF 720) for rearing (A) and for male escape rate (B).

Experiments were performed in climate-controlled chambers (KBWF (E6), model KBWF 720; Binder, GmbH, Tuttlingen, Germany). Egg batches aged 2–4 weeks, obtained from the above described mass-rearing colonies, were used for the trials. Prior to hatching (day 0), the hatch rate of the batch was determined. Eggs were then weighed following the egg quantification method described by Zheng *et al.* [49], to achieve a larval density of 3,600 first-instar larvae per tray, corresponding to 3.6 larvae/mL as specified in the FAO/IAEA *Aedes* mass-rearing guidelines [11]. On Day 1, approximately 20 hours post egg hatching, first-instar larvae were transferred to white plastic trays (dimensions: 408 × 252 × 82 mm), each containing 1 L of reverse osmosis water. The trays were placed within the climate chambers (Fig. S1A) set at five different constant temperatures (26 °C, 27 °C, 28 °C, 29 °C, and 30 °C) with fixed relative humidity at 80% and a photoperiod of 14:10 h (light-dark). Both *Ae. aegypti* and *Ae. albopictus* were reared simultaneously under identical environmental conditions. A HOBO data logger (ONSET HOBO data logger U12-012, Onset Computer Corporation, Bourne, MA, USA) was placed inside each climate-chamber to monitor conditions. Larvae were fed a 6% (w/v) IAEA diet consisting of 50% tuna meal, 35% black soldier larvae powder, and 15% brewer’s yeast, with a feeding regime that excluded days 2 and 3, implemented as follow: 40 mL on day 1, and 30 mL from day 4 to day 7 after egg hatching [26]. To minimize temperature fluctuations during feeding, the diet was pre-aliquoted into small cups and quickly dispensed during brief chamber openings. From day 6 to day 10, at 9:00 a.m. daily, tray contents were sieved quickly, with rearing water returned to their respective trays prior to pupal sorting using a Fay-Morlan glass sorter [12, 13]. Sorting for all trays was completed within one hour, after which larvae were immediately returned to their trays. Male and female pupae were manually counted daily and the following parameters were subsequently estimated:

i) Time to pupation, i.e., the period it takes for a mosquito larva to develop and reach the pupal stage after hatching from the egg. The mean time to pupation was calculated as follows:
$$\text{Mean time to pupation}=\frac{\sum \left(\text{days to pupation}\times \text{Number of pupae}\;\mathrm{on}\;\text{that}\;\mathrm{day}\right)}{\text{Total number of pupae}}$$
ii) Pupation rate, calculated as the ratio of total pupae collected to the initial number of larvae.
iii) Male escape rate; following sex-separation, approximately 105 male pupae collected at day 6 were kept in emergence cages (17.5 × 17.5 × 17.5cm, BugDorm-4, BD4M1515, MegaView Science Co., Ltd., Taichung, Taiwan) and provided with 10% sugar solution. Emerged males aged 3–4 days were transferred using mouth aspirator into an FTD version 1.1 and observed for 2 h to estimate escape rates, following the protocol described by Culbert *et al.* [9] and Maiga *et al.* [24].
iv) Adult body size; for each sex, temperature treatment, and experiment, ten adults were randomly taken. Their right wings were dissected for wing length measurement using a Dino-Lite Digital Microscope (Dino-Lite AMS7025X-USB Microscope, DinoEye Eyepiece cameras, New Taipei City, Taiwan) [39]. For each species, four technical repetitions were used per temperature treatment, with the entire experiment repeated three times.

### Effects of temperature and RH on escape rates during flight testing with the FAO/IAEA flight test device

For this experiment, *Ae. aegypti* was used. First-instar larvae were seeded at a density of 18,000 larvae per tray and fed a 4% (w/v) diet following the feeding regime and schedule previously described [24, 26]. Larvae and pupae were separated on day 6 post-hatching using a Wolbaki automatic sex sorter [15, 27]. Male pupae collected were kept in cages (30 × 30 × 30cm, BugDorm-1H; DP1000) with continuous access to 10% sugar solution. Adult males aged 5 to 6 days, were subsequently subjected to flight ability testing using the modified flight test device (FTD) version 1.1, as described by Maiga *et al.* [24]. The climate-controlled chambers (KBWF (E6), model KBWF 720) were configured as shown in Figure S1B to assess the effect of environmental factors.

For temperature assessment, five constant temperatures (15 °C, 20 °C, 26 °C, 30 °C, and 35 °C) were tested under a fixed constant RH of 65 ± 5%. These temperatures were selected to represent a broad spectrum of environmental conditions. While 26–30 °C corresponds to the optimal temperature range typically maintained in insectaries, the inclusion of lower (15–20 °C) and higher (35 °C) temperatures was intended to simulate suboptimal conditions that may arise in non-climate-controlled environments or during specific periods of the day, such as early morning, late evening, or midday. Mosquitoes were acclimated to each test temperature for a period of 5 minutes prior to flight testing. For RH evaluation, three levels (50 ± 5%, 65 ± 5%, and 80 ± 5%) were tested at a constant temperature of 26 °C.

Approximately 100 males were transferred using mouth aspirator to an FTD and observed for 2 h to evaluate their escape response. The experiment was conducted twice with four technical replicates for each temperature or RH treatment per trial.

### Statistical analysis

Data were analyzed using R Software, version 4.3.2 (R Development Core Team 2008) along with the RStudio, version 2024.10.31 environment (RStudio, Inc. Boston, MA, USA, 2016). Binomial generalized linear mixed models (GLMMs) fit by maximum likelihood (Laplace Approximation) were used, with pupation rate and escape rate as response variables, temperature and RH as fixed effects, and the replicate and experiment as random effects. A Gaussian linear mixed-effects model was used with time to pupation, male and female body size, assigned as response variables, temperature as a fixed effect, and replicate and experiment as random effects [21]. The full models were checked for overdispersion using Bolker’s function for validation.

## Results

### Effect of temperature on *Ae. aegypti* and *Ae. albopictus* development and quality

#### a) Time to pupation

Figure 1 shows the time to pupation for *Ae. aegypti* and *Ae. albopictus* reared at temperatures ranging from 26 °C to 30 °C. Time to pupation was significantly affected by temperature only at 30 °C, for which both species exhibited significantly faster development. However, we did not observe a significant impact of temperature on this parameter within the range 26–29 °C (*p* > 0.05). A 1 °C shift from 29 °C to 30 °C led to significant differences in time to pupation for both *Ae. aegypti* (df = 52, *t* = −2.455, *p* = 0.017 and *Ae. albopictus* (df = 52, *t* = −5.64, *p* < 0.001). Similarly, a 2 °C shift from 28 °C to 30 °C resulted in significantly shorter time to pupation for *Ae. aegypti* (df = 52, *t* = −2.64, *p* = 0.01) and *Ae. albopictus* (df = 52, *t* = −6.179, *p* < 0.001). Under these experimental conditions, *Ae. albopictus* tended to develop slightly faster than *Ae. aegypti*, although this difference was not statistically significant (df = 107, *t* = −0.81, *p* = 0.42).

**eFigure 1:**
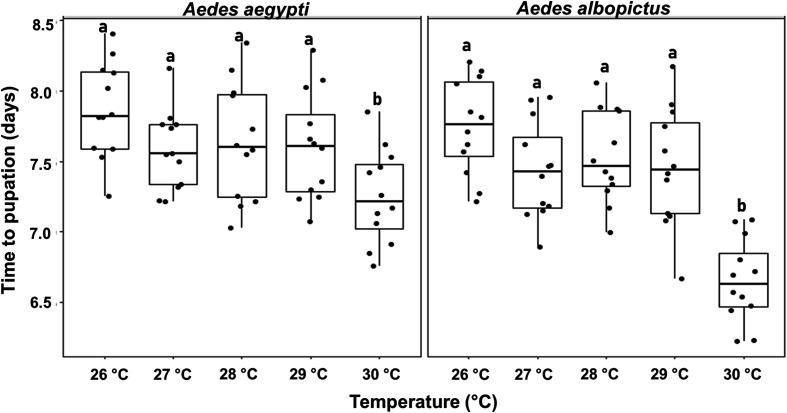
Time to pupation in *Aedes aegypti* and *Aedes albopictus* reared under different temperature conditions. The boxplots represent the median (line across the middle), quartiles (25th and 75th percentiles), and the minimum and maximum values (endpoints of the vertical lines). Jittered points represent individual replicates. Within each species, different letters between temperature treatments indicate statistically significant differences (LMMs, *p* < 0.05).

#### b) Pupation rate

**Figure 2:**
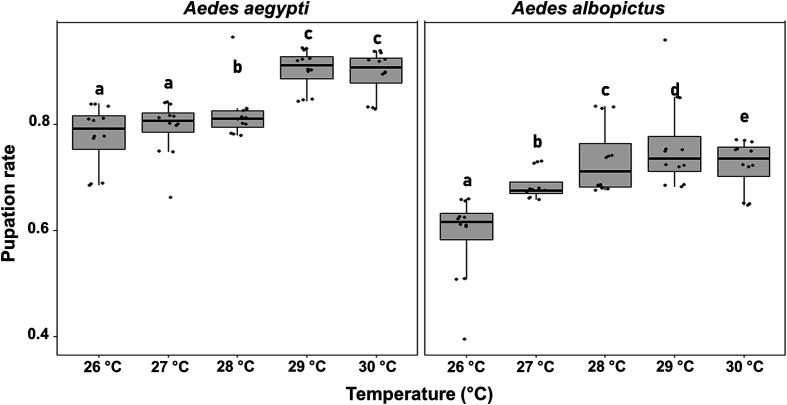
Pupation rates in *Aedes aegypti* and *Aedes albopictus* reared under different temperature conditions. The boxplots represent the median (line across the middle), quartiles (25th and 75th percentiles), and the minimum and maximum values (endpoints of the vertical lines). Jittered points represent individual replicates. Within each species, different letters between temperature treatments indicate statistically significant differences (GLMMs, *p* < 0.05).

The total pupation rate is shown in Figure 2. Overall, pupation rates increased with rising temperature in both *Ae. aegypti* and *Ae. albopictus*. However, under identical rearing conditions, *Ae. albopictus* exhibited significantly lower pupation rates compared to *Ae. aegypti (GLMM,* Z=-36.865, *p* < 2e–16). Compared to the reference temperature of 28 °C, all other tested temperatures resulted in significant differences in pupation rates in both *Ae. aegypti* and *Ae. albopictus* (GLMM, *p <* 0.05), indicating that even a 1 °C temperature shift significantly affected pupal yield. A change of only 1 °C in temperature resulted in a notable effect on pupal output, corresponding to a 5.5 ± 1.9 % difference in pupal production.

#### c) Escape rate

**Figure 3:**
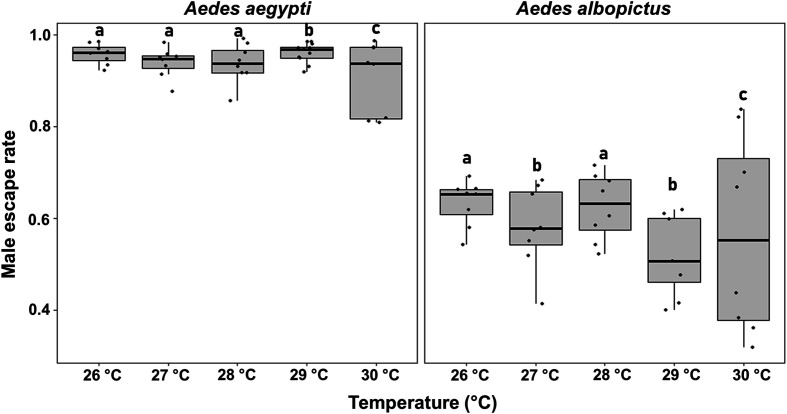
Male escape rates in *Aedes aegypti* and *Aedes albopictus* reared under different temperature conditions. The boxplots represent the median (line across the middle), quartiles (25th and 75th percentiles), and the minimum and maximum values (endpoints of the vertical lines). Jittered points represent individual replicates. Values were compared only between adjacent temperature treatments and different letters between adjacent treatments indicate statistically significant differences (GLMMs, *p* < 0.05).

The escape rate is presented in Figure 3. Each temperature treatment was compared to its adjacent temperature(s). Overall, rearing temperature significantly influenced male escape rates in both *Aedes* species, but in an unpredictable way. Across all tested temperatures, *Ae. albopictus* consistently showed significantly lower escape rates than *Ae. aegypti (z =* −15.055, *p* < 2e–16). A slight 1 °C temperature increase from 28 °C to 29 °C had a significant effect on male escape rates, resulting in an increase for *Ae. aegypti (*z = 2.39, *p* = 0.016) and a decrease for *Ae. albopictus (*z = −3.34, *p* < 0.05).

#### d) Body size

**Figure 4:**
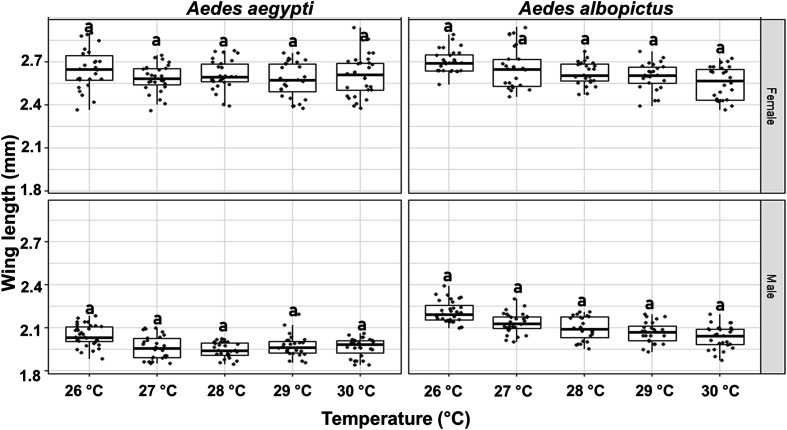
Body size of male and female *Aedes aegypti* and *Aedes albopictus* reared under different temperatures. The boxplots represent the median (line across the middle), quartiles (25th and 75th percentiles), and the minimum and maximum values (endpoints of the vertical lines). Jittered points represent individual measurements. Different letters between temperature treatments indicate statistically significant differences (LMMs, *p* < 0.05).

Female mosquitoes exhibited consistently greater wing length compared to males for both species. Male *Ae. albopictus* exhibited significantly larger wing length than *Ae. aegypti* males (df = 266, *t* = 14.269, *p* < 0.05), whereas no such significant differences were observed for females (df = 266, *t* = 1.627, *p* = 0.104). However, across all tested temperatures (26–30 °C), no significant differences in wing length were detected for males or females within either of the species (Figure 4).

### Effects of temperature and RH on escape rates during flight testing with the FAO/IAEA flight test device

**Figure 5:**
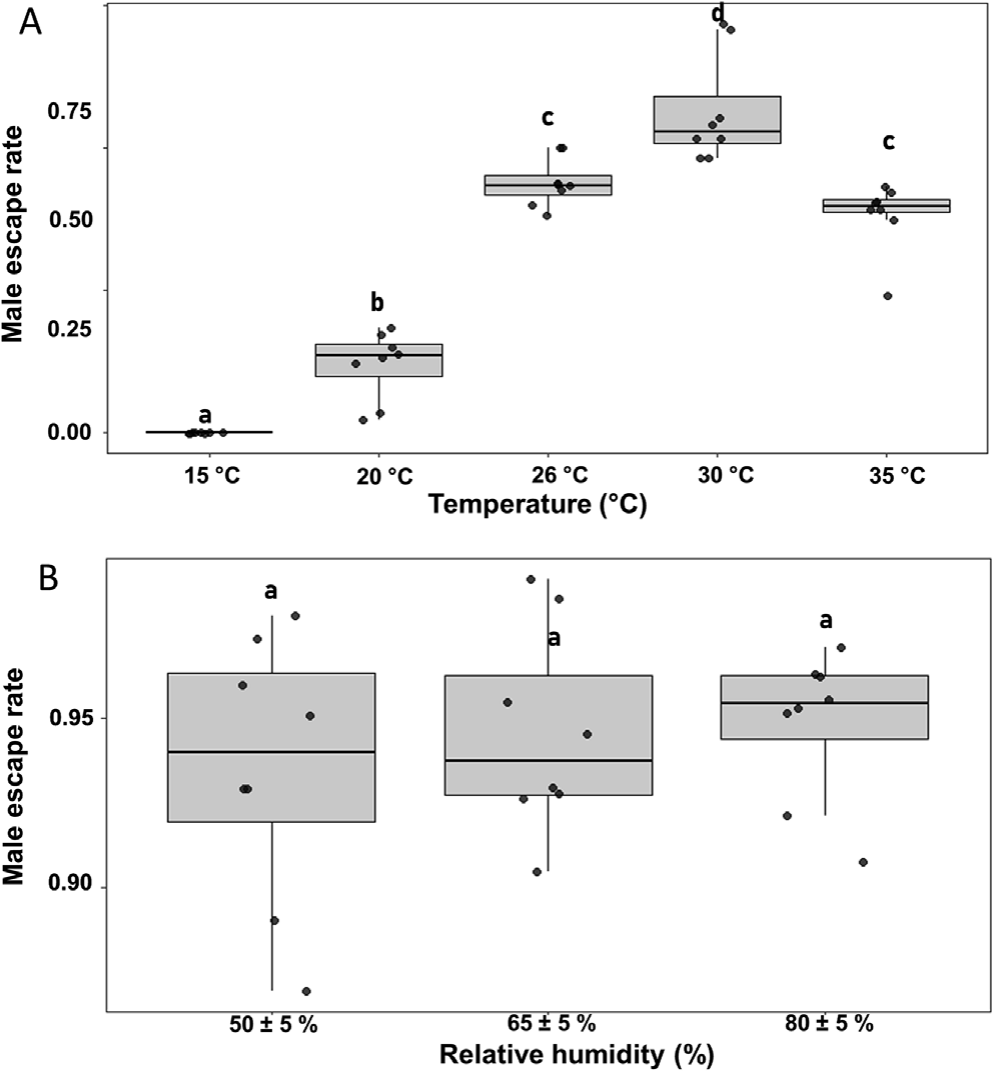
Male escape rates in *Aedes aegypti* evaluated using the FAO/IAEA flight test device across different temperature (A) and relative humidity (B) conditions. The boxplots represent the median (line across the middle), quartiles (25th and 75th percentiles), and the minimum and maximum values (endpoints of the vertical lines). Jittered points represent individual replicates. Different letters between treatments indicate statistically significant differences (GLMMs, *p* < s0.05).

The effects of environmental conditions (temperature and RH) on male escape rates during flight testing with the FTD are shown in Figure 5. Routine testing conditions, 26 °C and 65% RH, were considered to be the reference environment. Escape rates varied significantly across the range of tested temperatures (Figure 5A). At 15 °C, the escape rate was negligible, indicating very low flight activity at this temperature. Escape rates increased with temperature, reaching an optimal peak at 30 °C. At 35 °C, the escape rates declined, suggesting stress-related response. Relative to the reference temperature 26 °C, escape rates at all tested temperatures differed significantly (*p* < 0.05), except at 35 °C, which showed no statistically significant difference.

In contrast, male escape rates were consistently high across all tested humidity levels (50 ± 5%, 65 ± 5%, and 80 ± 5%), with no statistically significant differences observed among these conditions (Figure 5B).

## Discussion

Consistency in mosquito production and quality within mass-rearing settings, coupled with the accurate assessment of male escape rates using the FTD is essential for the effective planning and success of any male-based release programs, such as the SIT. Environmental factors, particularly temperature and RH, exert a profound influence on *Aedes* mosquito biology, behavior, and ecology. Temperature is a major driver of development rates with higher temperatures generally accelerating mosquito development [17, 40]. In *Aedes* species, development is known to occur within a temperature range of approximately 14 °C to 36 °C (Bar-Zeev, 1958), with optimal development reported between 25 °C to 30 °C for *Ae. albopictus* [10, 48]. Specifically, Delatte *et al.* (2009) [10] identified 29.74 °C as the optimum temperature for development in this species. Based on the previous findings, most laboratories apply a rearing temperature range of 26–30 °C for mass-rearing *Aedes* mosquitoes. Despite this well-established knowledge and available guidelines, mass-rearing facilities frequently observe variability in rearing outcomes. Furthermore, while the FTD serves as a rapid quality control tool to assess adult male flight ability, its efficiency remains sensitive to operational and environmental variables, and thus requires further standardization to ensure consistency across laboratories. A better understanding of how environmental conditions affect both rearing performance and quality assessment tools is essential for refining and harmonizing SOPs and ensuring the consistent assessment of sterile male quality.

Our study demonstrated that *Ae. aegypti* (Juazero strain) and *Ae. albopictus* (Rimini strain) are sensitive to small shifts in rearing temperature. A minor deviation of 1–2 °C from the target rearing temperature significantly accelerated or delayed development, altered pupation rates, and affected overall pupal yield and male escape rates. For instance, a 1 °C increase from 29 °C to 30 °C resulted in a significantly shorter time to pupation in both species, while deviations from the reference temperature of 28 °C consistently affected pupation outcomes. In *Ae. aegypti,* Sasmita *et al.* [38] similarly reported shorter pupation times and lower pupation rates at 28 °C and 32 °C compared to 25 °C, though this study evaluated a larger, 3 °C temperature shift, and reared in microplates. Our findings obtained under larval density and feeding regimes that reflect mass-rearing conditions, are particularly relevant to vector control strategies, such as the SIT, which depends on the large-scale production of high-quality mosquitoes under controlled environmental conditions. Variability in pupal yield due to temperature fluctuation hinder production planning, thereby disrupting the consistency and reliability of SIT operations. Although the experimental design characterized by constant temperatures, short timescales, and the use of laboratory strains does not directly simulate climate-change scenarios, the pronounced developmental sensitivity of *Aedes* mosquitoes to small-scale temperature variations observed under laboratory conditions may foreshadow how climate-driven warming could affect mosquito population dynamics, survival, and the effectiveness of vector-control interventions, such as the SIT. According to the Intergovernmental Panel on Climate Change (IPCC) [3], a global temperature increases of 1.5 °C is expected to have substantial ecological and epidemiologic consequences, particularly for temperature-sensitive species such as mosquitoes.

Regarding adult quality, the male escape rate was also significantly affected by small-scale temperature shift, with notable differences observed between 29 °C and 30 °C. Such temperature-induced reductions in escape rate could impair the mating competitiveness of released sterile males, ultimately reducing SIT efficacy. The escape rate, as measured by the FTD, is a rapid and practical tool for routine quality monitoring. It serves as a general indicator of male vigor, but should not be considered a standalone predictor of male performance in the field. In contrast, the wing length, an indicator of adult size and potential fitness, remained relatively stable across the tested temperature range (26–30 °C) for both mosquito species and sexes, suggesting that this morphological trait is less sensitive to minor thermal variation. These results are consistent with those of Shahrudin *et al*. [40] who reported no differences in wing size among male *Ae. albopictus* reared at constant temperatures from 20 °C to 35 °C. Similarly, Mohammed and Chadee [30] observed no variation in wing length in *Ae. aegypti* across temperatures between 25 °C and 30 °C.

Both species, *Ae. aegypti* and *Ae. albopictus* exhibited similar overall trends in response to temperature variation across the measured parameters. However, the magnitude of these responses differed between the two species. For instance, a 1 °C increase from 28 °C to 29 °C significantly shortened the time to pupation in both species, yet it resulted in a significant increase in escape rates in *Ae. aegypti*, and a significant decrease in *Ae. albopictus*. Also, *Ae. albopictus* consistently displayed significantly lower pupation and escape rates compared to *Ae. aegypti* under identical rearing conditions. Such interspecific differences have been observed in other contexts, such as their varying responses to water hardness [28]. Moreover, even within the same species, intra-species variability exists, particularly in relation with their behavioral activities, as shown in previous research [2, 47]. These findings highlight the importance of developing species/strains-specific temperature standards to optimize both rearing efficiency and adult quality in mass-rearing programs. Although development was accelerated at 30 °C, this temperature negatively affected mosquito quality indicators, including reduced male escape rates. These findings clearly demonstrate that a rearing temperature of 30 °C is suboptimal for both *Ae. aegypti* and *Ae. albopictus* mass-production programs.

For male-based release programs such as the SIT, it is essential to evaluate the quality of mass-reared males prior to release. The escape rate measured by the FTD [9] is a key quality parameter. To ensure consistency and reliability of measurements and standardize operating procedures, several factors have been assessed [24]. However, the effects of temperature and RH during FTD during testing remain unexplored. This study showed that variation in temperature (about 5°C shifts) during testing had a significant effect on escape rates of *Ae. aegypti* (Juazero strain), while RH levels ranging from 50% to 80% had no significant impact on flight performance, indicating that temperature is the primary environmental factor affecting the escape rate during FTD assessment. However, temperature and RH are interrelated parameters. It is not surprising that temperature fluctuations may alter the saturation deficit [5, 19]. Consequently, the potential effect of the saturation deficit cannot be completely ruled out because it may introduce a confounding effect when the effects of temperature are interpreted. Fortunately, relative humidity itself was not a significant factor in this study. As the temperature increased from 15 °C to 35 °C, the escape rates rose and peaked around 30 °C, then declined at higher temperatures. This trend is consistent with the general shape of a thermal performance curve, which describes how ectothermic organisms exhibit improved physiological and behavioral performance as temperature approaches an optimal range, followed by a rapid decline beyond that optimum. Given that mosquitoes are poikilothermic organisms, metabolic rate and muscle function are directly influenced by environmental temperature [34]. Their flight muscles rely on enzymatic processes that are temperature sensitive [41]. At lower temperatures, metabolic activity is reduced, resulting in diminished mobility, whereas excessively high temperatures can induce thermal stress that impairs muscle function. For instance, Rowley and Graham [35] observed maximal flight speed in female *Ae. aegypti* at 32 °C. Based on these findings, the temperature of the room where flight tests are conducted should be controlled, ideally maintained at a fixed reference temperature (with minimal variation) within the acceptable range between 26 °C and 35 °C to ensure consistent and reliable quality assessments.

One limitation of our study is that we used only one strain to assess the impact of the environmental parameters, whereas there may be intra-specific differences, for instance with *Ae. albopictus* strains from tropical areas. Although Maiga *et al.* [24] found no significant differences in the escape rate among three *Ae. albopictus* strains from Italy, Spain, and China, the temperature or RH-related variation in the escape rate observed in our study should not be generalized. Several studies have demonstrated that behavioral activity can vary between populations, in some cases even more than between species [2, 47]. In addition, this study did not include measurements of physiological or biochemical parameters, such as metabolic rate, energy reserves, stress markers, and flight muscle condition. Consequently, the mechanistic basis for the disproportionate effects of small temperature variations on quality metrics remains unclear. Future research will incorporate these physiological indicators to establish links between environmental stressors, quality parameters, and mosquito performance.

## Conclusion

This study demonstrated the sensitivity of mosquito biological traits to small-scale temperature changes, emphasizing the need for strict temperature control in both larval rearing environment and adult quality control using the FTD. For vector control programs using SIT or other biocontrol strategies, these results are critical to optimizing mosquito production and quality and ensuring overall program success.

## Data Availability

All data generated or analyzed during this study are included in this published article.

## Supplementary material

The supplementary material of this article is available at https://www.parasite-journal.org/10.1051/parasite/2026019/olm.
